# Success of microvascular surgery; repair mesenteric injury and prevent short bowel syndrome: a case report

**DOI:** 10.1186/1471-227X-7-11

**Published:** 2007-08-14

**Authors:** Unal Aydin, Omer V Unalp, Pinar Yazici, Adem Guler

**Affiliations:** 1 Department of General Surgery, Ege University School of Medicine, Izmir, Turkey

## Abstract

**Background:**

Superior mesenteric injury is a rare entity but when it occurs, short bowel syndrome is one of the uninvited results of the emergency surgical procedures.

**Case presentation:**

We present a 19-year-old boy with blunt abdominal trauma which caused serious mesenteric injury. Because ultrasound revealed free intraabdominal fluid, he underwent emergency laparotomy. Adequate vascularization of approximately 20 cm of proximal jejunal segment and approximately 20 cm of terminal ileum was observed. Nevertheless, the mesentery of the rest of the small intestine segments was ruptured completely. We performed an end-to-end anastomosis between a distal branch of the superior mesenteric artery in the mesentery of the ileal segment and a branch of the superior mesenteric artery using separate sutures of 7.0 monofilament polypropylene. The patient's gastrointestinal passage returned to normal on the postoperative day 2. He recovered without any complication and was discharged from hospital on the postoperative day seven.

**Discussion:**

In this case report, we emphasize the importance of preservation of injured mesenteric artery due to abdominal trauma which could have resulted in short bowel syndrome.

## Background

Although isolated small bowel or superior mesenteric artery (SMA) injury due to blunt abdominal trauma is quite rare, abdominal trauma is the reason of short bowel syndrome (SBS) in approximately 10% of the patients. Treatment and management of the SBS are difficult. SBS as a result of major intestinal resection due to isolated SMA injury was reported in the literature before. To our knowledge, this is the first case in the literature that the small intestine segment was preserved by arterial anastomosis to prevent SBS in a young patient with blunt abdominal injury. In this case report, we emphasized the importance of preservation of injured mesenteric artery due to abdominal trauma which could have resulted in SBS.

## Case presentation

**Figure 1:**
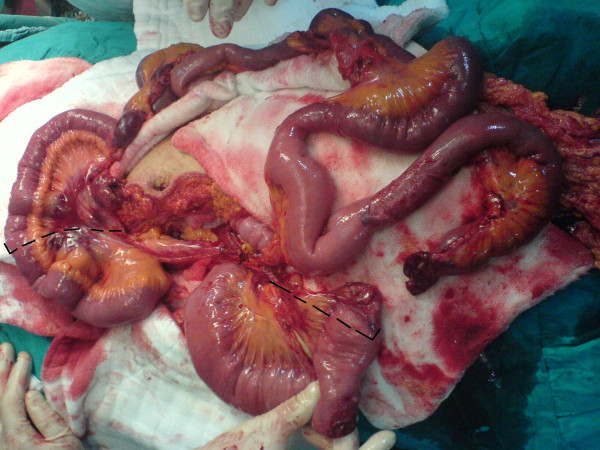
The ruptured small intestine and mesentery root (right part of the dashed lines).

**Figure 2:**
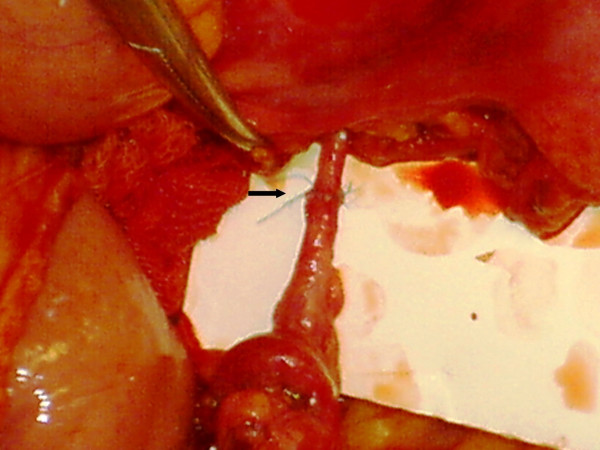
The anastomosis between the arteries using 7/0 polypropylene suture.

**Figure 3:**
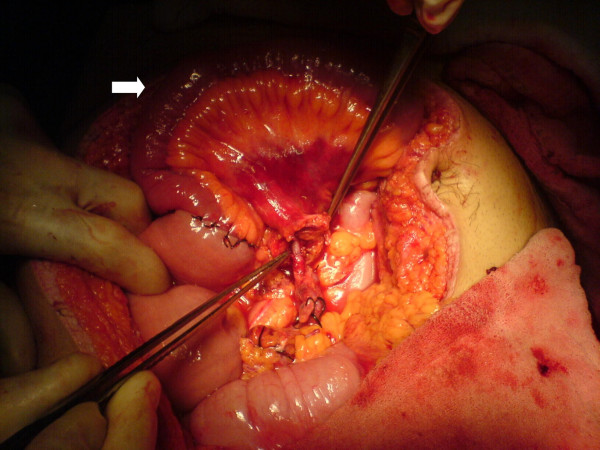
After reperfusion, the view of the small bowels in the early period. The arrow shows the ischemic ileal segments.

**Figure 4:**
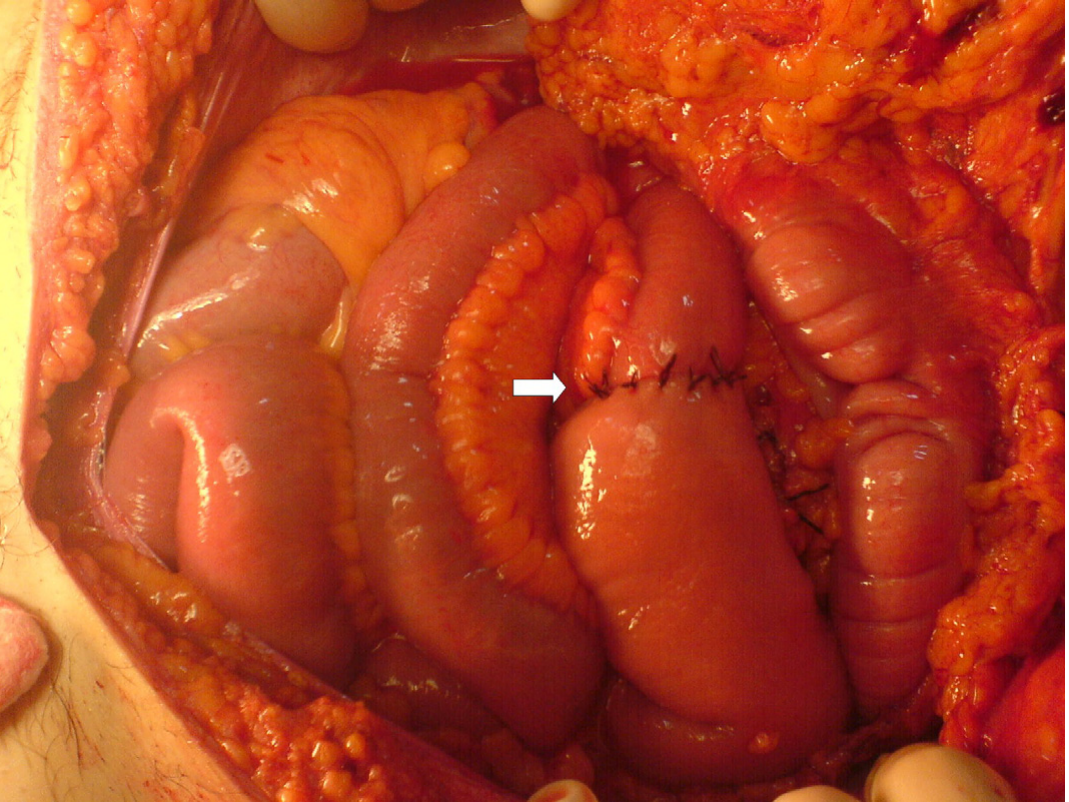
60 minutes after reperfusion. Double layered end-to-end small intestine anastomosis.

**Figure 5:**
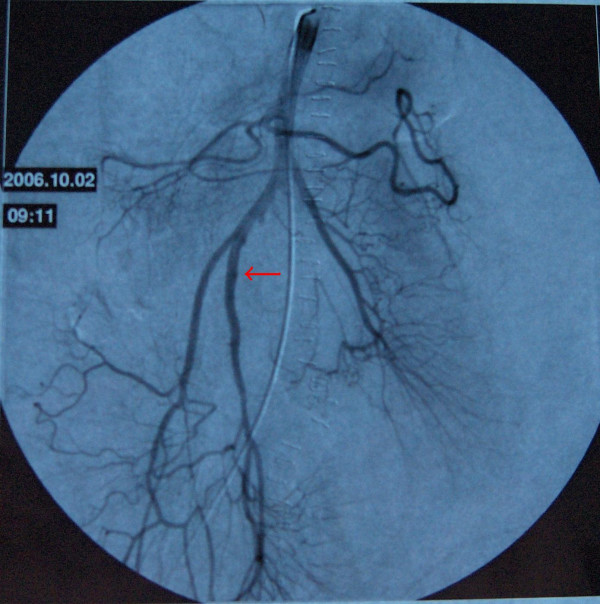
Mesenteric angiography performed on the postoperative first month. The arrow shows the anastomosis between the arteries.

19-year-old man was brought to the emergency service after a high speed car crash. On arrival at the emergency department, the patient was monitorized and maintained a heart rate of 120 beats/minute (sinus tachycardia), with a blood pressure of 90/40 mmHg. Fluid resuscitation was initiated with normal saline. There was no obvious exsanguination injury present. The Glascow Coma Scale score was 15. There was found no significant thoracic or cranial trauma. A Focused Abdominal Sonography for Trauma (FAST) examination revealed free intraperitoneal fluid in each quadrant of the abdomen. The patient was promptly transported to the operating room where he underwent exploratory laparotomy. Midline incision from processus xyphoideus to symphysis pubis was employed. Intra-operative observation revealed approximately 1000 cc of free blood in the peritoneal cavity. Liver, spleen and the other solid organs were normal through macroscopic examination, but small intestine and its mesentery looked extensively damaged. The vascular trunk between the inferior pancreaticoduodenal and right colic artery of the superior mesenteric artery was completely transected. Adequate vascularization of approximately 20 cm of proximal jejunal segment and 20 cm of terminal ileum were observed. Nevertheless, remainder of the mesentery of the small intestine was ruptured completely. Although the proximal mesenteric arcade was damaged, the root of the SMA was patent. There were also multiple perforations in the small intestine, and a severe hematoma located in the mesentery, 40 cm proximal to the ileocecal junction (see Figure 1). Unfortunately, the distal ileal segment to the hematoma was also seemed to be ischemic. After application of a bull-dog clamp to the root of the superior mesenteric artery, the bleeding could be taken under control. The hematoma in the mesentery of the ileal segment was evacuated. A distal branch of the superior mesenteric artery found in the hematoma was dissected and prepared for end to end anastomosis with a ruptured branch of superior mesenteric artery 2 mm in size. End-to-end anastomosis between the arteries was performed using interrupted 7.0 monofilament polypropylene (7/0 prolene) sutures after irrigation with heparinised saline (1 unit heparin/1 ml IV fluid) of both arteries (see Figure 2). During the operation, the patient was given 4 units of blood and 4 litres of volume-expansive and crystalloid solutions. After reperfusion, ischemic segments of the small intestine were resected and an end-to end double layer anastomosis between the jejunum and the ileum was performed by using 3/0 polydiaxonon and 3/0 silk (see Figure 3, 4). The patient's gastrointestinal passage returned to normal on the postoperative day 2. He did well and was discharged from hospital on the postoperative day 7. Until discharge, he had semi-liquid defecation four times daily. The patient was prescribed low molecular weight heparin for 7 days, starting on the postoperative day 1. On the last control, one month after the operation, the patient had no complaints. He used to have one defecation daily and fat analysis of the stool was in normal ranges. In the control mesenteric angiography, the vascular anastomosis was normal (see Figure 5).

## Discussion

Trauma to the small bowel accounts for less than 1% of all traumas [1-3]. Blunt trauma accounts for 33% of small bowel injuries and penetrating trauma accounts for 67%. Of those sustaining injury to small bowel, 93% require intestinal resection (4). Intestinal resection after traumatic injury may be due to the trauma to the mesenteric vessels and/or the bowel wall itself. Injury to the superior mesenteric artery (SMA) is an uncommon and devastating entity with mortality rates reported as high as 64% [5]. Exsanguinating hemorrhage is the main reason of early deaths. Late deaths usually are occurred secondary to sepsis, multiple organ failure, and the sequelae of ischemic bowel in those who survive their initial surgical procedure and SBS.

The common reasons of SBS in adults are; tumors such as desmoid tumor, ischemia due to thromboembolic events, intussusception or volvulus, Crohn's disease, malabsorption and motility disorders and traumatic injury of the bowel and its blood vessels. In the past, repetitive resections of small intestine in Crohn's disease were the main cause of SBS. As a result of the improvements of conservative and surgical therapy of Crohn's disease, SBS in adult patients now occurs more frequently due to vascular disorders (such as embolism/thrombosis of the superior mesenteric artery/thrombosis of the mesenteric veins) or intestinal strangulation (such as volvulus or incarceration). Rarely, in patients with trauma, extensive bowel resection is indicated. The small bowel has a large functional reserve capacity. Thus, resection of up to 50% of the small bowel is usually tolerated without any symptoms, and in most patients, resection of up to 50–70% leads to transient malabsorption, only. However, a residual length of the small bowel of less than 200 cm may result in SBS in the early postoperative period, and if less than 70–100 cm of small bowel are left almost all patients develop SBS. Moreover, almost all patients with less than 60 cm of small bowel need long-term parenteral nutrition [6]. The resection of the colon is crucial as the overall extension of bowel resection in deciding the severity of the symptoms of the SBS. Moreover, losses of the duodenum or the terminal ileum, particularly the ileocecal valve, impair absorption much more than loss of other parts of the small bowel. The duodenum and the ileocecal region have specific absorptive functions and play a crucial role in the regulation of postprandial gastrointestinal motility and secretion. These functions may not or only partly be replaced by other parts of the small bowel.

The usual mechanism of the mesenteric injury is direct crushing of the small bowel against the vertebral column [7]. Tearing and shearing forces, especially seat belts in car accidents, applied to the abdomen, particularly at points of mesenteric attachment, can also be the mechanism. According to the surgical literature, proximal jejunum and distal ileum are more prone to injury from blunt trauma because of the short mesentery in these areas [8].

Treatment modalities in short bowel syndrome can be classified as; dietary therapy, medical treatment, total parenteral nutrition, small bowel transplantation. The 5-year survival rate of isolated small bowel transplantation is 45%, and that of combined small bowel and liver transplantation is 37% [9]. Although enteral independency can be achieved in time, in about half of the cases, parenteral nutrition (PN) is indicated for irreversible and chronic intestinal failure [10,11]. By the usage of parenteral nutrition, long-term survival in patients with SBS can be achieved, but has associated morbidity and high expense. Prevention of this condition remains an important and challenging goal.

Conservative surgical approach to the mesenteric vascular structures and small intestine by using microvascular surgical techniques can help preventing the development of SBS in patients with mesenteric trauma. We preferred the anatomical anastomosis between the branches of the superior mesenteric arcade which were appropriate after preparing the damaged ends. Several choices of grafts, synthetic or autologous, are also possible to maintain the arterial patency. But due to limited time on this emergency operation, we firstly tried reconstruction of the mesenteric artery branches using advantages of easy mobilization of the mesentery. It did work fine and the patency of anastomosis as its original form was proved by angiography control. Thus, we didn't need to use any interpositional graft, which is generally unnecessary for intraabdominal anastomosis unlike extremity injuries. Revitalized small intestine segments by using microsurgery can be crucial in prevention of SBS in the postoperative period in patients with blunt trauma. Saving small intestine segment of 40 cm in our case prevented the SBS and served a better quality of life for the patient.

In conclusion, in appropriate cases, alternative conservative procedures including microvascular surgery should be considered when extensive small bowel resections are required because of mesenteric injury for preventing SBS.

## Competing interests

The author(s) declare that they have no competing interests.

## Authors' contributions

UA made substantial contributions to conception and design, acquisition of data and managed the operation.  OVU was involved in drafting the manuscript and acquisition of data.  PY was involved in drafting the manuscript and revising it critically for important intellectual content.  AG received the final approval of the version to be published.

## Pre-publication history

The pre-publication history for this paper can be accessed here:


